# Expert Involvement Predicts mHealth App Downloads: Multivariate Regression Analysis of Urology Apps

**DOI:** 10.2196/mhealth.5738

**Published:** 2016-07-15

**Authors:** Nuno Pereira-Azevedo, Luís Osório, Vitor Cavadas, Avelino Fraga, Eduardo Carrasquinho, Eduardo Cardoso de Oliveira, Miguel Castelo-Branco, Monique J Roobol

**Affiliations:** ^1^ Department of Urology Erasmus University Medical Centre Rotterdam Netherlands; ^2^ Urology Department Porto Hospital Centre Porto Portugal; ^3^ Urology Department Hospital Espírito Santo Évora Portugal; ^4^ Faculty of Health Sciences Beira Interior University Covilhã Portugal

**Keywords:** eHealth, mHealth, urology, mobile apps, new technologies

## Abstract

**Background:**

Urological mobile medical (mHealth) apps are gaining popularity with both clinicians and patients. mHealth is a rapidly evolving and heterogeneous field, with some urology apps being downloaded over 10,000 times and others not at all. The factors that contribute to medical app downloads have yet to be identified, including the hypothetical influence of expert involvement in app development.

**Objective:**

The objective of our study was to identify predictors of the number of urology app downloads.

**Methods:**

We reviewed urology apps available in the Google Play Store and collected publicly available data. Multivariate ordinal logistic regression evaluated the effect of publicly available app variables on the number of apps being downloaded.

**Results:**

Of 129 urology apps eligible for study, only 2 (1.6%) had >10,000 downloads, with half having ≤100 downloads and 4 (3.1%) having none at all. Apps developed with expert urologist involvement (*P*=.003), optional in-app purchases (*P*=.01), higher user rating (*P*<.001), and more user reviews (*P*<.001) were more likely to be installed. App cost was inversely related to the number of downloads (*P*<.001). Only data from the Google Play Store and the developers’ websites, but not other platforms, were publicly available for analysis, and the level and nature of expert involvement was not documented.

**Conclusions:**

The explicit participation of urologists in app development is likely to enhance its chances to have a higher number of downloads. This finding should help in the design of better apps and further promote urologist involvement in mHealth. Official certification processes are required to ensure app quality and user safety.

## Introduction

Medicine is constantly evolving, and medical research and development are greatly influenced by available and new technology. Mobile health (mHealth), defined as “the delivery of healthcare services via mobile communication devices” [1], is a new element of eHealth based on mobile phone and tablet apps. Apple and Google provide the leading mHealth platforms (iOS and Android, respectively), with over 160,000 medical apps between them [2]. The number of mHealth apps is expected to grow, not least because both companies have announced mHealth to be a top priority [3,4].

mHealth has had an impact in several medical specialties, including anesthesia [5], cardiology [6], and psychiatry [7]. Moreover, it has been applied to a diverse set of problems facing both health care professionals (HCPs) and patients, including apps that use augmented reality in the operating room [8], risk calculators for clinical practice [9], and digital diaries that aid in patient monitoring [10]. The apps available for urological practice were summarized in a recent review [11], which highlighted that not all urology apps share the same popularity; while some apps are downloaded very infrequently, other apps have been downloaded over 10,000 times. To date, the factors that contribute to the number of downloads of a medical app have not been characterized.

The economic literature indicates several factors that affect app downloads, with price being one of the significant predictors [12,13]. Even though some users are willing to pay for more sophisticated features in better-quality apps and see the price as a marker of quality, others only download free apps, sometimes with limited features. In fact, some users download a paid version only after trying the free version or use in-app purchases to get access to additional features. It has been shown that the option of in-app purchases can affect a user’s decision to download the app [12].

The exchange of opinions and experiences online, that is, online word-of-mouth, influences ecommerce sales [14]. Word-of-mouth has two main characteristics: volume (the total amount of word-of-mouth) and valence (whether the attitude is positive or negative). Word-of-mouth volume generates the cognitive consequence of awareness, while word-of-mouth valence produces the cognitive consequence of attitude [15]. In the mobile apps market, to predict the number of downloads, authors use the number of user reviews as the volume and the user rating as the valence [12].

Previous studies have shown that app demand decreases with the app file size. As apps become more complex they increase in size, meaning that they take longer to download and for users to try them. Moreover, they occupy additional space in the device memory [12,13]. App availability on both platforms (Apple App Store and Google Play Store) may raise awareness about the app, influencing the number of downloads [12].

The developer’s textual and visual description of an app can undoubtedly contribute to the willingness of users to download an app. Prior studies have shown that textual information and visual images affect consumer purchase decisions [16,17]. For mobile apps, the app description’s length and the number of screenshots significantly affect app demand [12].

Other factors that may positively influence the number of downloads are the app’s age (ie, how long the app has been available) and availability of updates (ie, whether the app has been updated since launch) because these are surrogates of the app’s evolution [12]. Availability of an update also raises awareness for the app because updates allow the app to be featured in the “New & Updated Apps” category of the Google Play Store. In contrast, age-restricted content in an app will have a negative impact on the number of downloads because it limits the number of potential users [12].

The delivery of mHealth in urology will, as in all medical fields, largely depend on app availability, benefits, and user friendliness. Although economic studies have identified some of the factors that influence app downloads [12-18], given the specificity of medical apps, we hypothesized that the involvement of a health care expert could be a significant determinant in the ultimate number of downloads of a urology app. Therefore, we aimed to determine the predictors of the number of urology app downloads, including the contribution made by HCP involvement.

## Methods

### Search Strategy

We conducted a commercial review of all urology apps for the Android mobile operating system in the Google Play Store (Google Inc, Mountain View, CA, USA) up to August 31, 2015: we examined all apps containing the term “urology” in their metadata (ie, the title, description, keywords, or version history). We included only urology-specific apps in this study; hence, we excluded apps containing content related to other medical specialties (ie, generic apps targeting multiple subjects; eg, an anatomy atlas), product advertisements (ie, apps only promoting pharmaceuticals or clinical equipment), and apps solely allowing the user to schedule private appointments.

We selected only Android apps for study because, in contrast to Google, Apple does not report the number of individual app downloads. Furthermore, Apple only lists the top 200 medical apps ranked by a nondisclosed proprietary algorithm. However, no urology apps were present in the top 200 medical apps listed in either app store.

### Predictor Variables for the Number of Downloads

For each app, 2 reviewers (NP-A and MR) recorded all available information according to 12 predetermined variables: (1) number of downloads, the dependent variable, (2) number of written user reviews, (3) price in euros, (4) average user rating (number of stars from 1 to 5), (5) app size (in megabytes), (6) number of screenshots (ie, an actual app image that showcased its features and functionality), (7) length of app description (number of characters in the app description, not including spaces), (8) app availability in the Apple App Store (ie, whether the app was available for iOS mobile phones or tablets), (9) new versions available (ie, whether the app had been updated since launch), (10) app age (number of days available in the Google Play Store), (11) absence of age restriction (ie, defined by the developer as having content appropriate for all ages), and (12) availability of in-app purchases (ie, the opportunity to buy extra content). Table 1 lists these variables and their descriptions. We did not download the apps.

**Table 1:** Variables included in the model to predict the number of downloads of urology apps.

Variables	Description
Level of downloads^a^	Level 0: no downloads
	Level 1: 1–5 downloads
	Level 2: 6–10 downloads
	Level 3: 11–50 downloads
	Level 4: 51–100 downloads
	Level 5: 101–500 downloads
	Level 6: 501–1000 downloads
	Level 7: 1001–5000 downloads
	Level 8: 5001–10,000 downloads
	Level 9: 10,001–50,000 downloads
No HCP^b^ participation	0: Other
	1: No HCPs mentioned
Other HCP participation	0: Other
	1: Other HCPs, pharmacists, and nurses
Urologist participation	0: Other
	1: Urologist or urological association participation
Number of reviews	Number of reviews in the Google Play Store
Actual price	Actual price of the app in euros
Average user rating	User evaluation on a scale from 1 to 5 stars
App size	App file size in megabytes
No age restriction	0: Age restriction
	1: No age restriction (ie, appropriate for all ages)
Number of screenshots	Number of screenshots in the Google Play Store
Length of description	Number of characters (without spaces) in the textual app description in the Google Play Store
Availability in the Apple App Store^c^	0: Not available
	1: Available
Version	0: One version
	1: New version exists
App age	Number of days available on the market
In-app purchases	0: No in-app purchase
	1: In-app purchase available

^a^The exact number of downloads is not available from the Google Play Store. We categorized it according to the system used by Google in the Play Store.

^b^HCP: health care professional.

^c^Available for iOS mobile phones or tablets.

To test the hypothesis that urologist involvement influences app downloads, we added a further variable to our model: HCP participation. We identified HCP participation by examining the app’s description and considered it to be present only when explicitly mentioned. We classified the participating HCP as urologist (ie, urologist or urological association), other HCPs (ie, other medical doctors, pharmacists, or nurses), or no HCP (ie, no explicit mention of an HCP). The 2 reviewers gathered download data based on the classification system of level of downloads used by Google in the Play Store (Table 1). At the time of final review (August 31, 2015), no urology apps had been downloaded over 50,000 times.

### Statistical Analyses

Analyses were performed using IBM SPSS Statistics v20 (IBM Corp). We considered *P*<.05 to be statistically significant in all analyses. Descriptive analyses and multivariate ordinal logistic regression identified the factors predicting app downloads.

## Results

A total of 250 Google Play apps contained the term urology in their metadata. We excluded 121 apps: 109 were generic apps (ie, not designed specifically for urology, eg, ArchieMD 3D Health: PREVIEW), 11 were for making appointments (eg, Dr Fateh Singh Appointments), and 1 app was designed solely for product advertisement (Actient Pharmaceuticals).

Of the 129 included apps (Multimedia Appendix 1, Multimedia Appendix 2), 90 (69.8%) were free. Of the paid apps, the prices ranged from €0.68 (Urology Glossary) to €83.15 (The 5 Minute Urology Consult 3), with an average price of €8.45. The average app rating was <3 stars (mean 2.65), and 92 (71.3%) had no written review. There were 5 screenshots per app on average, and the length of the description varied from 3 to 3348 characters (without spaces). The number of days since publishing varied from 1 to 1733 (average 721 days) (Table 2).

**Table 2:** Summary descriptive statistics for continuous variables for apps containing the term urology.

		Mean	SD	Range	Median
Number of reviews		0.84	2.08	0–12	0
**Actual price (**€)					
	All apps	2.55	9.89	0–83.15	0
	Paid apps	8.45	16.68	0.68–83.15	2.69
Average user rating (no. of stars)		2.65	2.13	0–5	3.5
App size (MB)		7.37	10.36	0.01–48	3.2
Number of screenshots		5.4	3.86	1–25	4
Length of description (nonspace characters)		896.08	872.24	3–3348	531
App age (days)		721.18	425.76	1–1733	699

**Figure 1:**
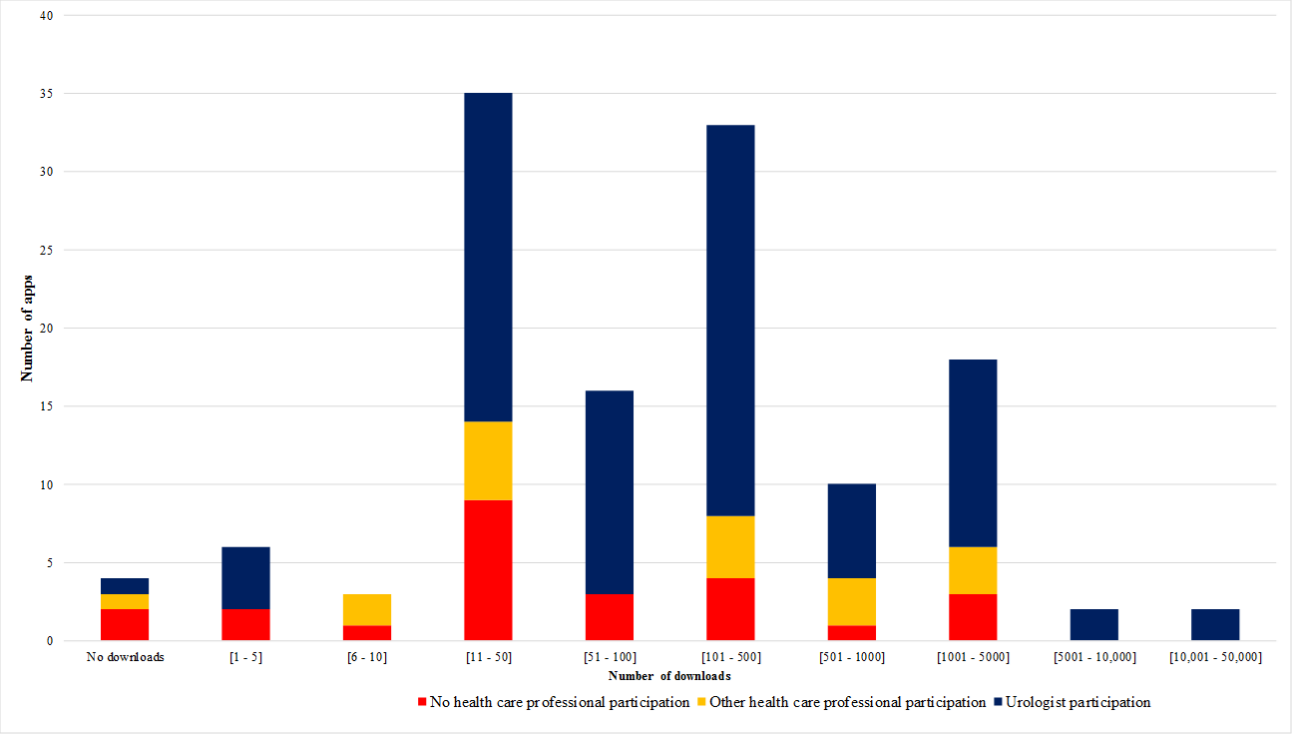
Number of urology apps per level of downloads and health care professional participation.

Figure 1 shows the number of apps in each level of downloads and HCP participation. The proportion of apps with HCP participation was greater in the higher levels of downloads. Moreover, in the 2 highest levels (>5000 downloads), only apps designed with the participation of urological experts were present.

Even though 2 (1.6%) apps had >10,000 downloads (level 9), half of all urology apps had ≤100 downloads (level 4 or less). At the time of this review, 4 apps (3.1%) had not been downloaded (Table 3).

**Table 3:** Frequencies for the categorical and binary variables.

		Frequency	Percentage	Cumulative percentage
**Level of downloads**				
	0: no downloads	4	3.1	3.1
	1: 1–5 downloads	6	4.7	7.8
	2: 6–10 downloads	3	2.3	10.1
	3: 11–50 downloads	35	27.1	37.2
	4: 51–100 downloads	16	12.4	49.6
	5: 101–500 downloads	33	25.6	75.2
	6: 501–1000 downloads	10	7.8	82.9
	7: 1001–5000 downloads	18	14.0	96.9
	8: 5001–10,000 downloads	2	1.6	98.4
	9: 10,001–50,000 downloads	2	1.6	100
**No HCP** ^a^ **participation**				
	Other	104	80.6	80.6
	No HCPs mentioned	25	19.4	100
**Other HCP participation**				
	Other	111	86.0	86.0
	Other HCPs, pharmacists, and nurses	18	14.0	100
**Urologist participation**				
	Other	43	33.3	33.3
	Urologist or urological association participation	86	66.7	100
**No age restriction**				
	Age restriction	72	55.8	55.8
	No age restriction	57	44.2	100
**Availability in Apple App Store**				
	Not available	36	27.9	27.9
	Available	93	72.1	100
**Version**				
	One version	66	51.2	51.2
	New version exists	63	48.8	100
**In-app purchases**				
	No in-app purchase	118	91.5	91.5
	In-app purchase available	11	8.5	100

^a^HCP: health care professional.

Although most apps, that is, 86 of 129 (66.7%), were developed with specialist urological input and other HCPs were involved in a further 18 apps (14.0%), 25 apps (19.4%) had no documented HCP involvement. A total of 57 apps (44.2%) had no age restriction. Only 11 apps (8.5%) had in-app purchases available.

Multivariate logistic regression revealed the factors contributing to urology app downloads (Table 4). Apps developed with urologist involvement were more likely to be installed than those without expert involvement (*P*=.003). Availability of in-app purchases (*P*=.01), a higher user rating (*P*<.001), and a higher number of written reviews (*P*<.001) were also significantly associated with app downloads. The app price was inversely related to the number of downloads (*P*<.001). The other evaluated factors (app age, app size, absence of age restriction, number of screenshots, length of description, availability in the Apple App Store, and new published versions) were not significantly associated with app downloads. The Nagelkerke *R*^2^ statistic, which measures the strength of the association between the dependent variable and the predictor variables, was satisfactory.

**Table 4:** Multivariate ordinal logistic regression of factors contributing to number of urology app downloads.^a,b^

Variables	Estimates^c^	SE	*P* value	95% CI
App age	0.001	.0004	.24	–0.0003 to 0.001
Other HCP participation	0.469	.602	.44	–0.71 to 1.65
Urologist participation	1.43	.479	.003	0.49 to 2.37
Number of reviews	0.440	.102	<.001	0.24 to 0.64
Actual price in euros	–0.071	.020	<.001	–0.11 to –0.03
Average user rating	0.337	.089	<.001	0.16 to 0.51
App size	0.018	.017	.30	–0.02 to 0.05
No age restriction	0.498	.346	.15	–0.18 to 1.18
Number of screenshots	0.047	.048	.33	–0.05 to 0.14
Length of description	–0.0004	.0002	.09	–0.001 to 0.00006349
Availability in the Apple App Store	–0.641	.441	.15	–1.5 to 0.22
Version	–0.372	.340	.27	–1.04 to 0.29
In-app purchases	1.67	.682	.01	0.33 to 3.0
Nagelkerke *R*^2^			.48	

^a^The dependent variable is the level of downloads.

^b^The reference level for health care professional (HCP) participation is “No HCP participation.”

^c^Estimates are the ordered log-odds regression coefficients and they show the relative magnitude (ie, relative impact of the factor) and direction (ie, positive or negative) of the impact of the listed variables on the level of downloads.

## Discussion

### Principal Findings

The lack of studies on the predictors of the number of downloads for medical apps in the PubMed database suggests that this is the first study of its kind in mHealth. However, economic studies determined the predictors of downloads for generic apps, which we tested in this study. We showed that inexpensive apps developed with expert urological input and with optional in-app purchases were more likely to be installed. Furthermore, apps with higher user ratings and with a larger number of written user reviews were more likely to have a greater level of downloads. These results confirmed, for the first time, that urologist participation in app development positively influences urology app downloads.

Although the availability of various medical apps has been thoroughly documented, the factors that predict their downloads have, until now, not been studied. Given that mHealth is a rapidly evolving and novel field, these data are useful for practitioners and app developers interested in developing urology apps. Furthermore, the data are important for mHealth policy makers and regulators because no best practice guidelines exist with respect to medical app development.

mHealth is still a relatively new concept, and its full potential has yet to be fully explored. The number of downloads of mHealth apps will depend not only on available technologies, but also on the apps and their safety, effectiveness, and usability. However, concerns have been raised about medical apps, namely their scientific accuracy and user security [5,7], which are exacerbated by the lack of regulation. The level of regulation should be proportional to the degree of clinical implication derived from the app, ranging from low (eg, apps that give access to online medical journals, which only show content that has already been peer reviewed) to high (eg, apps that dispense clinical advice).

Apps have the potential to be hazardous to uninformed users, either by error, such as miscalculation when using an opioid dose calculator [19], or by making false claims, such as dermatology apps that claim to diagnose skin cancer in spite of evidence that they misclassify 80% of textbook melanomas [20] and apps that guarantee to cure breast cancer [21]. Although these concerns have attracted the attention of public entities such as the European Union, which has published a green paper on mHealth [22], and the US Food and Drug Administration, which has issued some nonbinding suggestions [23], there is still no mandatory certification for mHealth apps. To address the lack of official guidelines, urological societies could participate in the regulatory process by publishing mHealth recommendations similar to those issued for social media [24-26]. In this way, app safety and accuracy can be improved by the involvement of medical experts at the early stages of app development and by promoting peer review.

Our results confirm our initial hypothesis that the explicit participation of an expert in urology in the app development process increases its chances to be downloaded. Given the lack of external certification of mHealth apps, one possible explanation for this result is that users are reassured to know that a health care specialist collaborated in the app design. Expert involvement could be equivalent to a “quality mark,” guaranteeing that the app is safe and scientifically valid. However, users must be aware that, because there is no official way to authenticate the veracity or the extent of the expert participation, unscrupulous developers could potentially misuse this approach via deceptive advertising or false endorsement. Interestingly, however, our findings also indicated that there is still a deficit of HCP participation in urology app development, with only two-thirds of apps having expert participation. This is consistent with previous reports on expert involvement in app development in other disciplines, perhaps signifying a wider trend across mHealth that needs to be addressed [27,28].

Cheaper apps with optional in-app purchases were associated with a greater level of downloads. As with mobile game users, mHealth users seem to prefer to pay less initially but to have the opportunity to buy additional benefits, features, or functionalities via in-app purchases, rather than paying a higher upfront price [12]. An app’s chance of having a higher number of downloads also increased with a higher number of reviews or average user rating, which is consistent with other fields in which published reviews have been shown to affect the choices of new users [12,16]. Although customer reviews were a significant determinant of downloads, they were lacking in most apps, making it harder for potential users to learn about the app without purchasing it themselves. To ameliorate this issue, developers should provide comprehensive details about the app in their description.

A systematic review has shown that eHealth adoption by HCPs is dependent on multiple factors, namely the involvement of users in the development and implementation phases, ease of use, demonstrated advantages of the system, and adequate training and support [29]. The security of the eHealth system was the most important factor in the acceptance of eHealth by patients [30].

Even though, in the generic mobile market, factors such as app size, number of screenshots, length of description, app age, availability in other mobile stores, availability of new versions, and absence of age restriction have a significant impact on the number of downloads, we found that it was not the case in urology apps. Further studies are needed to determine whether this trend is specific to urology apps or also happens in other medical fields.

Future research may consider the number of positive or negative reviews as a potential factor to predict app downloads. It should also focus on what types of urological apps and what segments of this specific market (ie, patients, HCPs, or both) have higher downloads. Furthermore, subsequent investigations should compare the number of downloads of urological apps with those in other medical fields in order to gain insights into the state of mHealth.

### Limitations

This study has some limitations. We limited our commercial review of urology apps to the search term “urology.” We included only urology-related apps and collected app data solely from information available in the Google Play Store and developers’ websites. Nevertheless, the Google Play Store and developers’ websites are the main sources of information available to potential new users before downloading the app; therefore, our study mimics the real-life information available to the user before purchase.

Android leads the mobile phone market with over 80% of market share, and there are more apps available in the Google Play Store. This is in part explained by the 2 platforms’ different approval processes: iOS apps have to undergo a thorough review process developed by Apple, but Android apps are immediately published online [3]. This distinct method may also influence the quality of the apps, which could be the subject of further research. We were unable to perform a similar analysis for the Apple App Store because Apple does not disclose the number of app downloads, instead only listing the top 200 medical apps calculated using their proprietary algorithm. However, we noted no urology apps in the top 200 medical apps listed in either the Apple App Store or the Google Play Store at the time of data collection. Other mobile app platforms make up <5% of the overall market share [31] and, at the time of our research, no urology apps were available in the Blackberry Mobile Market and only 3 urology apps were available in the Microsoft Store Marketplace; the numbers of downloads of these apps were not publicly available.

Displayed information about the level of downloads in the Google Play Store can in itself influence downloads: if a user has to choose between 2 similar apps, most of the time they will download the most popular app first. For the sake of clarity, we studied only explicit expert participation, and it is possible that some app developers consulted medical experts during app design but did not mention it; there is, therefore, a risk of misclassification for this variable. However, when medical involvement was reported, there was no objective way to determine the extent of participation. The lack of a standardized format for the disclosure of expert participation and the absence of readily available tools to quantify it requires further study and future recommendations.

### Conclusions

To our knowledge, this is the first study to determine predictors of urology app downloads. The explicit participation of urologists in app development is likely to enhance its chances of having a greater number of downloads. Furthermore, in-app purchases, cheaper apps, and those with higher user ratings and number of written reviews are more likely to have more downloads. Until a regulated approval process is implemented by government health authorities, analogous to the one that exists for medical devices, two pragmatic changes to urology mHealth app publishing could promote user safety and assure content quality: first, medical apps should include a full disclosure, similar to that provided in scientific papers; and second, urological societies could be involved with certifying the scientific integrity of mHealth apps by issuing a professional, peer reviewed app quality mark or standard. The efforts of the Health on the Net Foundation to guide users toward trustworthy health information online were justified by the results of 10th HON survey [32].

## Appendix

Multimedia Appendix 1 List of included apps.

Multimedia Appendix 2 Included apps ordered by the number of installs.

## Abbreviations

HCP: health care professional

## References

[1] Torgan C (2009). The mHealth Summit: Local & Global Converge.

[2] Reseach2Guidance (2015). mHealth App Developer Economics 2015: The Current Status and Trends of the mHealth App Market.

[3] (2016). HealthKit: Develop Health and Fitness Apps That Work Together.

[4] (2015). Google Fit: The Google Fit SDK.

[5] Kraidin J, Ginsberg SH, Solina A (2012). Anesthesia apps: overview of current technology and intelligent search techniques. J Cardiothorac Vasc Anesth.

[6] Martínez-Pérez B, de la Torre-Díez I, López-Coronado M, Herreros-González J (2013). Mobile apps in cardiology: review. JMIR Mhealth Uhealth.

[7] Shen N, Levitan M, Johnson A, Bender JL, Hamilton-Page M, Jadad AR, Wiljer D (2015). Finding a depression app: a review and content analysis of the depression app marketplace. JMIR Mhealth Uhealth.

[8] Rassweiler J, Rassweiler M, Müller M, Kenngott H, Meinzer H, Teber D, ESUT Expert Group (2014). Surgical navigation in urology: European perspective. Curr Opin Urol.

[9] Roobol M, Azevedo N (2014). The Rotterdam prostate cancer risk calculator: improved prediction with more relevant pre-biopsy information, now in the palm of your hand. http://www.jurology.com/article/S0022-5347(14)01780-7/pdf.

[10] Pepper J, Zhang A, Li R, Wang XH (2015). Usage results of a mobile app for managing urinary incontinence. J Urol.

[11] Pereira-Azevedo N, Carrasquinho E, Cardoso de Oliveira E, Cavadas V, Osório L, Fraga A, Castelo-Branco M, Roobol MJ (2015). mHealth in urology: a review of experts' involvement in app development. PLoS One.

[12] Ghose A, Han SP (2014). Estimating demand for mobile applications in the new economy. Manage Sci.

[13] Telang R, Garg R (2011). Estimating App Demand From Publicly Available Data.

[14] Davis A, Khazanchi D (2008). An empirical study of online word of mouth as a predictor for multi-product category e-commerce sales. Electronic Markets.

[15] Liu Y (2006). Word of mouth for movies: its dynamics and impact on box office revenue. J Marketing.

[16] Decker R, Trusov M (2010). Estimating aggregate consumer preferences from online product reviews. Int J Res Marketing.

[17] Ghose A, Ipeirotis PG, Li B (2012). Designing ranking systems for hotels on travel search engines by mining user-generated and crowd-sourced content. Marketing Sci.

[18] Sinkinson M (2012). The Determinants of Supply and Demand for Mobile Applications: Working Paper #12-27.

[19] Haffey F, Brady RR, Maxwell S (2013). A comparison of the reliability of smartphone apps for opioid conversion. Drug Saf.

[20] Wolf JA, Moreau JF, Akilov O, Patton T, English JC, Ho J, Ferris LK (2013). Diagnostic inaccuracy of smartphone applications for melanoma detection. JAMA Dermatol.

[21] Mobasheri MH, Johnston M, King D, Leff D, Thiruchelvam P, Darzi A (2014). Smartphone breast applications: what's the evidence?. Breast.

[22] European Commission (2014). Green Paper on Mobile Health (“mHealth”).

[23] U.S. Department of Health and Human Services, Food and Drug Administration (2015). A Mobile Medical Applications Guidance for Industry and Food and Drug Administration Staff.

[24] Rouprêt M, Morgan TM, Bostrom PJ, Cooperberg MR, Kutikov A, Linton KD, Palou J, Martínez-Piñeiro L, van der Poel H, Wijburg C, Winterbottom A, Woo HH, Wirth MP, Catto JW (2014). European Association of Urology (@Uroweb) recommendations on the appropriate use of social media. Eur Urol.

[25] Matta R, Doiron C, Leveridge MJ (2014). The dramatic increase in social media in urology. J Urol.

[26] American Urological Association Social Media Best Practices.

[27] Carter T, O'Neill S, Johns N, Brady RW (2013). Contemporary vascular smartphone medical applications. Ann Vasc Surg.

[28] Visvanathan A, Hamilton A, Brady RW (2012). Smartphone apps in microbiology: is better regulation required?. Clin Microbiol Infect.

[29] Gagnon M, Desmartis M, Labrecque M, Car J, Pagliari C, Pluye P, Frémont P, Gagnon J, Tremblay N, Légaré F (2012). Systematic review of factors influencing the adoption of information and communication technologies by healthcare professionals. J Med Syst.

[30] Chhanabhai P, Holt A (2007). Consumers are ready to accept the transition to online and electronic records if they can be assured of the security measures. MedGenMed.

[31] IDC Analyze the Future (2016). Smartphone OS Market Share, 2015 Q2.

[32] Pletneva N, Cruchet S, Simonet M, Kajiwara M, Boyer C Results of the 10th HON Survey on Health and Medical Internet Use.

