# Supplementary material for: Expert Involvement Predicts mHealth App Downloads: Multivariate Regression Analysis of Urology Apps
Source: JMIR Mhealth Uhealth. 2016 Jul 15;4(3):e86. doi: 10.2196/mhealth.5738 (PMC4967182; doi:10.2196/mhealth.5738)
Supplement: Multimedia Appendix 2 [file mhealth_v4i3e86_app2.pdf]

**App name**	**Level of success**	**HCPp**
SMARTfiches Urologie Free	10001-50000	HCP_Urology
Urology - Medical Dictionary	10001-50000	HCP_Urology
AUA Guidelines at a Glance	5001-10000	HCP_Urology
My Urologist - Мой уролог (ru)	5001-10000	HCP_Urology
APps en Urologia	1001-5000	No HCPp
AUA 2014 Annual Meeting	1001-5000	HCP_Urology
AUA Core Curriculum Mobile	1001-5000	HCP_Urology
AUAUniversity	1001-5000	HCP_Urology
Bladder Pal	1001-5000	HCP_Urology
DutasT	1001-5000	Other HCPp
EAU Stockholm 2014	1001-5000	HCP_Urology
EAU15	1001-5000	HCP_Urology
IURO Pelvic Floor	1001-5000	HCP_Urology
PI-RADS Prostate MRI	1001-5000	Other HCPp
Prostate Aerobics	1001-5000	No HCPp
Prostate Cancer Calculator	1001-5000	No HCPp
Prostate Pal 2	1001-5000	HCP_Urology
Renal & Urology News	1001-5000	Other HCPp
Uro+	1001-5000	HCP_Urology
Urology Board Review Manual	1001-5000	HCP_Urology
Urology Guidelines PrimaryCare	1001-5000	HCP_Urology
UrologyMatch	1001-5000	HCP_Urology
101st of Annual Meeting (Japan Convention Services, Inc第101回日本泌尿器科学会総会 Mobile Planner (Chinese)	501-1000	HCP_Urology
AUA Medical Student Curriculum	501-1000	HCP_Urology
AUA Men's Health Checklist	501-1000	HCP_Urology
IURO Andrology	501-1000	HCP_Urology
IURO Oncology	501-1000	HCP_Urology
Kidney Cancer	501-1000	No HCPp
Mictionary	501-1000	Other HCPp
PI-RADS IRM Prostate fr	501-1000	Other HCPp
Renal Mass - Bosniak	501-1000	Other HCPp
USICON 2014	501-1000	HCP_Urology
102nd Japanese UA (Japan Convention Services, Inc 第102回日本泌尿器科学会総会 Mobile Planner (Chinese)	101-500	HCP_Urology
64th Conference of the Urological Association - 제64차 대한비뇨기과학회 학술대회(Korean)	101-500	HCP_Urology
ATlas Quirurgico de Urologia Pratica	101-500	HCP_Urology
AUA Member Search	101-500	HCP_Urology
Bedwetting solutions	101-500	No HCPp
Besins UroMedica	101-500	Other HCPp
Braz J Urol	101-500	HCP_Urology
Briganti Nomogram	101-500	No HCPp
CBU 2013	101-500	HCP_Urology
CFU 2014	101-500	HCP_Urology
DGU 2012	101-500	HCP_Urology

App name	Level of success	HCPp
DGU 2013	101-500	HCP_Urology
DGU 2014 - Kongress App	101-500	HCP_Urology
EAU Pocket Guidelines	101-500	HCP_Urology
European urology	101-500	HCP_Urology
Int'l Urogynecology Journal	101-500	HCP_Urology
JUA2012	101-500	HCP_Urology
Masaüstü Başvuru Kitabı	101-500	HCP_Urology
Neurology advisor	101-500	HCP_Urology
Practical Urology	101-500	HCP_Urology
Prostate Cancer - ال برو سد تات سرطان (Arabic)	101-500	Other HCPp
Prostate In Focus	101-500	Other HCPp
PROSTATE INTERNATIONAL	101-500	HCP_Urology
ROU – РОУ (ru)	101-500	HCP_Urology
SIU	101-500	HCP_Urology
SIU 2013	101-500	HCP_Urology
SMU 2014	101-500	HCP_Urology
ÜCD Kongre 2012	101-500	HCP_Urology
UroAssist	101-500	No HCPp
URoCongress 2012	101-500	HCP_Urology
Urolithiasis Assist	101-500	No HCPp
Urología Práctica	101-500	HCP_Urology
Urological Ultrasound	101-500	Other HCPp
BMC Urology	51-100	HCP_Urology
EAUN Stockholm 2014	51-100	HCP_Urology
EAUN15	51-100	HCP_Urology
ESPU 2012	51-100	HCP_Urology
ESPU 2013	51-100	HCP_Urology
Fertilidad del varon	51-100	HCP_Urology
IURO Kidney	51-100	HCP_Urology
IURO Prostate Pro	51-100	HCP_Urology
Men's Guide To Prostate Health	51-100	No HCPp
NMIBC Toolbox	51-100	No HCPp
Tiempo de doblaje de PSA	51-100	No HCPp
Urología Para Ginecólogos	51-100	HCP_Urology
Urología Práctica para Médicos de Familia y Internistas	51-100	HCP_Urology
URologidagarna2012	51-100	HCP_Urology
Urology Nation	51-100	HCP_Urology
USICON 2013	51-100	HCP_Urology
28 Congreso de urologia 2014	11-50	HCP_Urology
AUA Journals	11-50	HCP_Urology
BAUS 2015	11-50	HCP_Urology
Bedwetting Diary	11-50	No HCPp
CAU2014	11-50	HCP_Urology
CMU 2015	11-50	HCP_Urology
CURE-UAB 2014	11-50	HCP_Urology
Daily-P	11-50	HCP_Urology
Foundation Urology	11-50	Other HCPp

App name	Level of success	HCPp
HapPee Time	11-50	No HCPp
IReflux Risk Calculator	11-50	No HCPp
ItsaMANTHING - Prostate Cancer	11-50	No HCPp
IURO Andrology PRO	11-50	HCP_Urology
IURO Oncology Pro	11-50	HCP_Urology
IURO Pelvic Floor Pro	11-50	HCP_Urology
JUS - Journal of Urological Surgery	11-50	HCP_Urology
Kidney And Bladder Problems	11-50	No HCPp
Learning Urology Quiz	11-50	Other HCPp
Mi prostata	11-50	HCP_Urology
Oxford Handbook Urology 2nd Ed	11-50	HCP_Urology
PipiTrainer Lite	11-50	Other HCPp
Prac. Urology for Primary Care	11-50	HCP_Urology
Prostate Cancer	11-50	Other HCPp
Prostate Health	11-50	HCP_Urology
Rotterdam Prostate Cancer Risk	11-50	HCP_Urology
SMARTfiches Urologie	11-50	HCP_Urology
Stop Your Child's Bedwetting	11-50	No HCPp
SuperFoods For Prostate Health	11-50	No HCPp
Uro Challenge	11-50	HCP_Urology
URologidagarna 2013	11-50	HCP_Urology
Urology For Gynecologists	11-50	HCP_Urology
Urology Glossary	11-50	No HCPp
Urology times	11-50	HCP_Urology
Urology, 1000 MCQs	11-50	Other HCPp
UroVote	11-50	No HCPp
Male impotence risk evaluation	6-10	No HCPp
Urology - Pediatric, 1000 MCQs	6-10	Other HCPp
Vasectomy Reversal	6-10	Other HCPp
100% Urologie	1-5	HCP_Urology
Dealing with Prostate Cancer	1-5	No HCPp
ProstateMD	1-5	HCP_Urology
The 5 Minute Urology Consult 3	1-5	HCP_Urology
Urological Surgery	1-5	HCP_Urology
Urology News	1-5	No HCPp
101 Tips Stop Child Bedwetting	no installs	No HCPp
Prostate Cancer v2	no installs	HCP_Urology
Testicle pain, testicle tumors	no installs	No HCPp
Urologic Nurse CURN, 800 MCQs	no installs	Other HCPp
